# The role of identity in the experiences of dementia care workers from a minority ethnic background during the COVID‐19 pandemic: A qualitative study

**DOI:** 10.1111/hex.13772

**Published:** 2023-05-05

**Authors:** Mishca Hughes, Sarah Butchard, Clarissa Giebel

**Affiliations:** ^1^ Department of Primary Care and Mental Health University of Liverpool Liverpool UK; ^2^ Mersey Care NHS Trust Liverpool UK; ^3^ NIHR Applied Research Collaboration North West Coast Liverpool UK

**Keywords:** care homes, Covid‐19, grounded theory, identity, minority ethnic groups

## Abstract

**Background:**

Care home staff working during the COVID‐19 pandemic experienced higher levels of stress and increased workloads. People from diverse ethnic backgrounds were disproportionately affected by the COVID‐19 pandemic. This study explored the identity experiences of care home staff from diverse ethnic backgrounds in the context of working during the COVID‐19 pandemic.

**Methods:**

Fourteen semistructured interviews were conducted between May 2021 and April 2022 with ethnic minority care home staff in England, who worked during the pandemic. Participants were recruited using convenience and theoretical sampling. Interviews were conducted via telephone or online platforms. A social constructivist grounded theory methodology was utilised in analysing the data.

**Findings:**

Participants described five key processes which facilitated or hindered the impact that their experiences had on their identity: dealing with uncertainty and transitioning into a COVID‐19 world; difficult emotions; experiences of discrimination and racism; the response from the care home and societal systems; and the personal vs collective responsibility. When participants' physical and psychological needs went unmet by support structures within the care home and/or society, they experienced a sense of injustice, lack of control and being unvalued or discriminated against by others.

**Conclusions:**

This study highlights the importance of recognising the unique needs of staff from diverse ethnic backgrounds working in care homes and adapting working practices to improve impact on identity, job satisfaction and staff retention.

**Patient and Public Involvement:**

One care home worker was involved in developing the topic guide and helping to interpret the findings.

## 1 INTRODUCTION

Currently, 416,000 people live in UK care homes.^1^ Staff face physical and emotional challenges, including low wages and poor working conditions.^2^, ^3^, ^4^, ^5^, ^6^ Heavy workloads and pressure to work when sick are also common.^7^ Government procedures during the Covid‐19 pandemic such as discharging untested patients and blanket ‘Do Not Attempt Resuscitation’ orders were criticised as human rights violations and worsened existing structural inequalities and challenges for staff.^8^ Staff struggled with understaffing, high workloads, challenging behaviour from residents and families^9^ and an inability to follow pandemic‐related protocols such as social distancing,^10^ which led to burnout and worsened mental health.^11^, ^12^

People from diverse ethnic backgrounds are overrepresented in the adult social care workforce (Skills for Care, 2020), and care home staff from diverse ethnic backgrounds reportedly experienced more discrimination during the COVID‐19 pandemic, encountering higher levels of racism, inadequate access to patient and public engagement (PPE), and adverse mental health outcomes related to COVID‐19 compared to their White colleagues. Staff can experience a lack of support from managers and colleagues relating to the incidence of discrimination, resulting in reduced positive social networks and structural disadvantages such as feelings of being undervalued, lack of trust in their organisations to handle harassment and bullying, which can harm their wellbeing and career growth.

Slay and Smith^13^ define professional identity as the beliefs and values that shape a professional's self‐concept. Training, experience and socialisation shape the professional identity of care home staff, affecting job satisfaction and the quality of care provided. Social Identity Theory^14^ views identity as fluid and derived from group membership, where intergroup differences and comparisons shape a person's sense of self. People from diverse ethnic backgrounds who identified less with ingroups or perceived discrimination due to their ethnicity had a greater fear of COVID‐19 and poorer mental health.^15^ Intersectionality theory recognises the interconnected nature of social identities and systemic inequalities that sustain disparities between groups.^16^, ^17^, ^18^ Challenges, such as low pay, high workload and a lack of recognition, negatively impact identity formation for care home staff, with additional challenges faced by staff from a diverse ethnic background, including discrimination, limited opportunities for advancement and lower pay, leading to undervaluation and marginalisation, but the cultural background may enhance their professional identity.

To describe ethnic minority groups, terms such as BAME (Black, Asian, and Minority Ethnic) and BME (Black and Minority Ethnic) have been used but have received criticism for reinforcing perceptions of homogeneity and maintaining the default of whiteness.^19^, ^20^ As a result, the term Staff of the Global Majority (SoGM) will be used in this paper, adapted from Ahsan's^21^ research.

This study aims to explore how the pandemic has impacted the identity of SoGM, who are currently underrepresented in research on the topic. Understanding the challenges faced by this group can help enhance support and improve the quality of care provided to residents in care homes.

## 2 METHODS

### 2.1 Participant recruitment

The inclusion criteria comprised that participants aged 18 years or older, self‐identified as belonging to an ethnic minority group, directly work with older adult care home residents during the pandemic in roles such as support worker, nurse or management, and proficiency in the English language for effective participation in the interview and the ability to provide informed consent. Exclusion criteria involved working in other care homes settings such as learning disabilities or mental health services.

A wide recruitment strategy was used, including care homes, community groups and social media. The researcher contacted National Institute for Health Research and The Enabling Research in Care Homes for additional support and care, and home managers were contacted via email or in person. Participants contacted the researcher, received an information sheet and were screened against the criteria.

Sampling methods included convenience and theoretical sampling, with the first five participants recruited through convenience sampling and the subsequent five using theoretical sampling to ensure a wide range of perspectives were heard, including senior positions. The final four interviews were conducted to refine the model and test hypotheses.

### 2.2 Data collection

**Figure 1:**
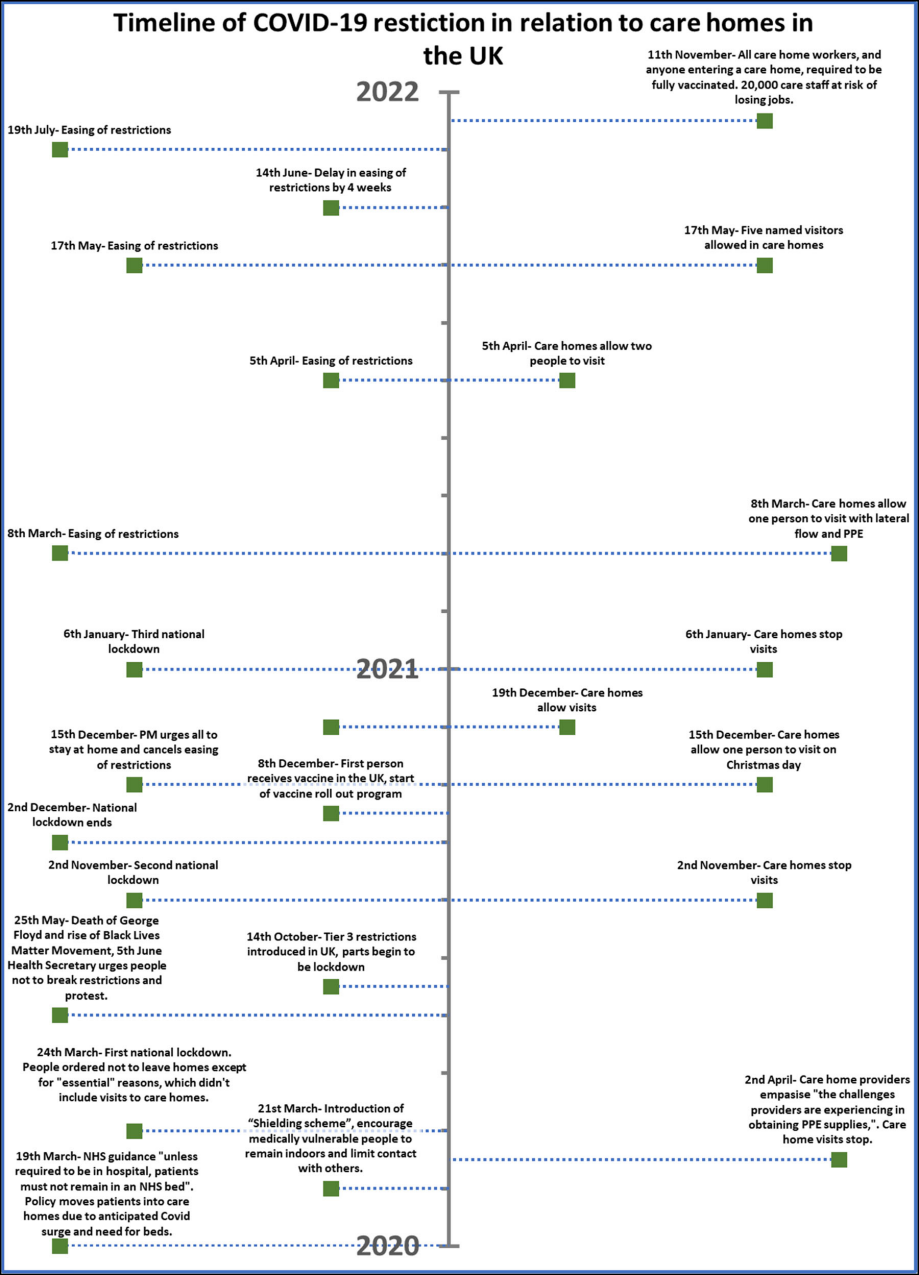
Timeline of COVID‐19 public health restriction in the United Kingdom, from March 2020 to November 2021. The left side represents UK restrictions, and the right side represents care home responses in relation to restrictions.

Ethical approval (approval number: 8133) was obtained from the University of Liverpool Ethics Committee before the study began in May 2021. Semistructured interviews were conducted between May 2021 and March 2022 via telephone or Zoom due to COVID‐19 restrictions. A timeline was developed to consider the social context of experiences (see Figure 1). Participants verbally confirmed their consent before interviews, which ranged from 20 to 72 min and were guided by flexible interview guides. A topic guide was piloted with the first two sets of five participants and refined iteratively during data collection to explore emerging categories such as discrimination to ensure it was relevant and adjusted for the final four interviews. Data collection was discontinued when data saturation occurred, and participants received a £10 shopping voucher.

### 2.3 Data analysis

The data were analysed using Social Constructionist Grounded Theory.^22^ Grounded theory was chosen as it can explore social relationships and behaviours where there has been little previous exploration and can include contextual factors of participants.^23^ The constructivist grounded theory approach focuses on how participants construct meaning in relation to their experiences and appreciates that existing literature, prior knowledge and experience of the researcher can all be used throughout the research process, which differs from other grounded theory approaches.^24^, ^25^ The interaction between the researcher and participants is also part of the process with emphasis on reflexivity throughout.^26^ Given the fast‐changing context the COVID‐19 pandemic presented to SoGM, Charmaz's constructivist grounded theory approach was deemed appropriate to choose due to its reflexive approach and ability to capture changing narratives.

Transcripts were externally transcribed, anonymized and analysed using NVivo 12. The lead researcher read through all transcripts multiple times, transcribed two interviews, and completed a summary of each interview to outline key categories and hypotheses.

Transcripts were coded using line‐by‐line coding and memos to create focused and theoretical codes. An initial theoretical model was drafted and tested by asking questions based on hypotheses from previous interviews. The model was edited four times as new codes and categories were constructed from the data. Participants were invited to provide feedback on a final draft of the model via email, but no responses were received.

### 2.4 Reflexivity

Reflexivity, emphasised by Charmaz,^22^ was important in this qualitative research, with the researcher considering their own experiences, and understanding of literature and acknowledging the dynamic nature of the research process. An initial reflective statement was written before data collection to outline the researcher's background, experience, views and expectations.

The researcher sought regular supervision to broaden their reflections, enhance objectivity and reliability, and monitor the impact of participant narratives. A research diary was kept to observe the researcher's values and experiences, which can influence the interpretation of data, and ensure the theoretical model was grounded in participant accounts for ecological validity.

### 2.5 Public involvement

The research team worked with a local care home and ethnic minority organisation community through the supervisors' connections. They involved a public advisor from an ethnic minority background who had previously worked in care to co‐design the study, formulate and validate questions, and provide feedback. The study materials were adapted for accessibility and understanding based on feedback from the advisor. The topic guide was created in collaboration with the public advisor.

## 3 RESULTS

A total of 14 participants took part in the study. Ten participants identified as female with the remaining four identifying as male. The participant's mean age was 42 years old (±1 year) (range 22–62 years), with the mean length of time working in a care home 10.5 years (±0.75), (ranging from 7 months to over 25 years). Seven of the participants' job roles within the care homes were support or senior support workers. A further three participants were nurses, two participants worked as team leaders and two were care home managers. Three participants identified as Black Caribbean, three as Black African, three as South Asian, two as Romani gypsy, one as Arab and one as Austronesian.

Overall, eight of the participants had no Covid‐19 related death of residents in their care home, with two participants reporting one death, two participants reporting two deaths, one participant reporting four deaths and the final participant reporting more than 10 deaths. Half of the participants in this study had contracted Covid‐19 while working in the care homes. This data guided further sampling and data collection as the diverse experiences of participants were sought and used to provide a context when interpreting findings.

Using grounded theory, three key categories were identified: universal experiences, physical and psychological needs met or unmet and the impact on identity. The following narrative explores the three key categories and associated subcategories in more detail, using quotes from participant interviews to highlight the findings.

### 3.1 Category 1: Universal experience

All participants described similar experiences when dealing with the changes that the pandemic cause to their work, emotions and identity. The following subcategories highlight the universal experiences described and the influencing process that determined whether participants felt their physical and psychological needs were met or not.

#### 3.1.1 Dealing with uncertainty and transitioning into a Covid‐19 world

All participants spoke about the impact and challenges they experienced due to the fast‐changing public health measures, guidance on infection control and restrictions on visitations. All participants described having additional responsibilities and roles due to the pandemic. This included providing more activities for residents and maintaining morale and hygiene standards.

it honestly used to feel really draining but then it came to a point where it's so natural now and I just feel like why did I struggle so much at that time. P4 (Female, Support worker)

#### 3.1.2 Difficult emotions

All participants reported experiencing difficult emotions due to working during the pandemic including, low mood, anxiety, loneliness and feeling physically and psychologically exhausted. Many participants also spoke about these difficult emotions impacting their mental and physical health but also an unspoken pressure to cope.

A lot of the staff were depressed, fed up, couldn't cope but because they needed the job, they just tried to stick it out. P11 (Female, Senior support worker)

#### 3.1.3 Discrimination

Many participants expressed that they faced discrimination from others in a variety of forms in the workplace and society due to their race, class, and professional level. Six participants described incidents of racism and discrimination, which could be encountered in many forms. Some participants spoke about incidents occurring on an interpersonal level at work, such as prejudicial statements, bullying and harassment.

a member of staff here turned around to a Black member of staff and said, Black people should be thankful to be in this country. P7 (Female, Team leader)

Some participants spoke about microaggressions from others in the workplace and community. These microaggressions consisted of chronic daily hassles, comments and avoidance from people, which resulted in a sense of not being welcome in spaces and marginalisation.

they speak to you as if you've just come out of nursing school. But they think because you're foreign and you're black and you work in this field they can treat you anyhow. P5 (Female, Nurse)

Participants also spoke about finding it difficult to adjust to knowing others around them held racist beliefs, at times not trusting their own judgement of others. Participants linked these interpersonal experiences of racism to societal structures such as government policies and the media. These experiences added an increased sense of being isolated and marginalised within society and the workplace and appeared to affect the way some participants felt about their workplace and the people they worked with.

I've worked with her (perpetrator of racist incident) for quite a while and all the time now and I'm thinking God is that what she's really thought of me you know … it was really degrading, and it was hard to come to terms with. P6 (Female, Care home manager)

#### 3.1.4 Response from the care home and societal systems

The presence, or lack, of support received from care homes and other societal systems, appeared to determine whether participants' felt their physical and psychological needs were met or not which in turn impacted their identity. Some participants explained they, and others within the care home, created policies and guidelines, supportive meetings and other interventions to meet the needs of staff and residents. Participants' identity was primarily influenced by their felt sense of justice, control, being valued and how personally responsible they felt for their and other experiences.

it's amazing to have the team support, the manager support, the head office support… I actually saw that if you have a good team, it doesn't matter what you are facing … the team is the main thing in the crisis. P12 (Female, Deputy manager)

### 3.2 Category 2: Physical and psychological needs met/unmet

All participants attempted to cope with or alleviate difficult emotions through strategies that aimed to meet their psychological needs. Physical and psychological support from others provided a protective factor for participants, increasing the likelihood of them feeling a sense of justice, control and value. Participants described attempting to access support through care home organisations, unions, communities, and friends. In contrast, when participants' psychological needs were not met by support structures within the care home and/or society, they experienced a sense of injustice, lack of control and being unvalued by others.

#### 3.2.1 Justice

Participants expressed mixed experiences of how incidences of discrimination were handled by management and unions which impacted the way participants perceived these experiences and on their identity. Participants that experienced a sense of justice and action after a single incidence of racism spoke about a feeling of shock and anger, ‘yeah that kind of floored me’ P7 (Female, Team leader). Some participants described strong action being taken to prevent other incidences of discrimination instigating a sense of being valued and protected. Participants described feeling more comfortable and confident that their experiences of racism and discrimination would be taken seriously if there was diversity within the team and management or if they had seen previous incidents dealt with appropriately.

I will not have it (racism) at this workplace … So, I had a staff meeting and I made it very clear my feelings on everything … because I wanted to protect my other care, residents, and my other workers… and made it perfectly clear that I will not stand for any kind of racism or anything on this unit. P6 (Female, Care home manager)

In comparison, participants that did not access support or got a sense of systemic injustice expressed that they felt unvalued or unprotected by others. Participants described accepting racist experiences as part of their work and world ‘I don't bother with it anymore, I think it's normal’ P5 (Female, Nurse), subsequently normalised it as part of the job. Some participants spoke of finding alternative ways to cope with frequent experiences of racism, including ignoring it, pushing difficult emotions away, and avoiding particular people and situations. These experiences appeared to negatively impact participants' sense of identity, outlining the fear and exhaustion they experienced being from a minority background.

it's terrifying to be Black, because of how the world is and how people think … The racism, the judgement, the name calling, it's tiring. It's exhausting. P7 (Female, Team leader)

Participants also described the fear and mistrust that this inaction created. They expressed a reluctance to report incidents of racism for fear of repercussions such as further incidents, worsening already challenging relationships and dynamics. This further impacted their feeling of isolation and marginalisation within work.

you don't feel comfortable discussing it with people who claim that they're on your side. Because eventually it gets right back to the people that give you these problems and it will be worse in a more polished way. So, you don't say anything. P5 (Female, Nurse)

#### 3.2.2 Control

Many participants expressed that when they felt a sense of choice and collaboration at work such as what shifts they worked or developing new strategies as a team they gained a sense of control and autonomy within the workplace. Participants reported that some workplaces and managers actively encouraged them to be part of decisions and changes to workplace strategies such as having open meetings when developing new workplace strategies.

having a meeting which covers mechanism that discuss and solve these problems altogether … a meeting you use to get the most effective way to get through Covid … to work more effectively as a team. P10 (Male, Care home manager)

In contrast, some participants expressed that they felt they lacked control over their work, environment and in some cases their own bodies. This sense of being controlled or pressured by others such as the government, media and individuals was demonstrated when participants described their frustration regarding the mandatory vaccination policies and the added pressure due to financial and family responsibilities.

I wasn't born in this country, so I have families back home I have to support, I help them financially. So, I had no choice … to me the government has taken away all my human rights. P7 (Female, Team leader)

#### 3.2.3 Value

Participants described feeling valued and protected by others as having a positive impact on their experiences, emotional resilience and identity. Interpersonal support offered by others (friends and family, colleagues and managers, their faith and other community groups) created a sense of shared connection and identity. Care home management demonstrated their valuing of staff through emotional support and other forms of protection such as an adequate supply of PPE. Many participants spoke about accessing support from their colleges enhanced a sense of belonging, closeness and acceptance.

talking to each other helped, and I think it's just being there for each other… a lot of the staff got a lot closer… they've got a new kind of respect now. P6 (Female, Care home manager)

Participants also described this increased sense of being valued when others in society attempted to show their recognition and appreciation, this was in various ways including clapping for them when they arrived home, positive comments and acts of appreciation such as making food for them.

In contrast, some participants expressed the lack of PPE and acknowledgement from others contributed to the sense of not being valued, protected or prioritised. This undervaluing was committed by the government, care home and the public and highlighted the disparity between the care home and the National Health Service (NHS) for some participants. This led to comparisons between the roles that people did and the perceptions that some professionals were prioritised and more valued than others.

#### 3.2.4 Responsibility

Participants described a tipping point between feeling personally and/or collectively responsible for the consequences related to working during the pandemic and this having an impact on their overall experiences. An enhanced capacity to balance personal and collective responsibility appeared to increase participants' ability to attend to their own emotional needs with self‐care or a forgiving mindset of ‘I am only human’ P1 (Male, Support worker). A level of personal responsibility supported a feeling of accountability and acknowledgement of their own limitations.

you're trying to do the best job you can. P6 (Female, Care home manager)

In contrast, some participants described experiencing too much personal responsibility regarding the consequences of Covid‐19 in their workplace (e.g., responsible for others' behaviours or solely responsible for an outbreak of Covid‐19 at the care home) in turn resulting in them blaming themselves or being self‐critical.

it just felt like I didn't really do anything much you know coming to the end and I was really devastated. P5 (Female, Nurse)

When participants were able to recognise the balance between collective and personal responsibility, they expressed an increased ability to incorporate areas that were not in their control (e.g., lack of PPE and appropriate guidelines) and reject their inner critic and gain a sense of being part of a system rather than just an individual.

It's crazy intense. You don't feel good about it and not because you're not doing a good job but because the feeling of it is rubbish because you can't because you don't have what you need … like more staff and PPE. P1 (Male, Support worker)

### 3.3 Category 3: Impact of identity

The impact participants' experiences had on their personal and professional identity was influenced by whether they felt their physical and psychological needs were supported by others.

#### 3.3.1 Increased pride in self

Participants that had their psychological needs met by support structures within the care home and/or society described an enhanced positive impact on their personal and professional identity and sense of self. Participants spoke about gaining a sense of being adaptable and skilled as a professional, which was reflected in their own sense of inner strength, pride and resilience as part of their identity. This increased pride also appeared to be linked to participants' sense of being a valued contributing member of society, giving them increased confidence and self‐esteem.

you just feel that you made a difference today. You feel that your work is important … I just feel useful to society. … I feel good about myself, and I feel good that I can actually do the job well. P2 (Female, Support worker)

Some participants described choosing to take pride in their ethnic and religious identity when they were supported and accepted in the workplace. For some participants, this sense of increased value, autonomy, and justice motivated them to engage in collective action, setting up committees and other support groups within the care home. Participants reported a motivation to support other SoGM, reduce feelings of isolation and had an increased ability to take pride in their multiple identities.

we have to be proud of who we are regardless of peoples difference … Not to be ashamed of who you are, I think that's very important, and we should be able to live the life that we want in a safe environment. P6 (Female, Care home manager)

#### 3.3.2 Diminished view of self

All participants described the link between them feeling a lack of control, justice and being unvalued by others and a negative impact on their professional identity and their role within society. Many participants described how their view of themselves had changed due to this sense of being unvalued as professionals and their inability to complete their roles adequately.

its crazy intense, you don't feel good about it (job) and not because you're not doing a good thing but because the feeling of it is rubbish, like the actual emotional stress of doing it is rubbish. P1 (Male, Support worker)

The ‘pressure to cope’ P11 (Female, Senior support worker) participant experiences could be internal or external and when they were not able to meet these expectations, they felt overwhelmed, exhausted and burnt out. Some participants spoke about the impact this had on their personal lives and identities, feeling unaccustomed or unsure of their personal selves.

You basically were like shells of themselves, it just piled up. P3 (Male, Support worker)

The negative experiences participants had impacted their views of others and the world. Some participants described trusting others less which added to their feeling isolated and marginalised. This all led to participants feeling a reduced self‐worth and self‐esteem.

100% never ever felt valued … but you didn't have a feeling of self‐worth, they made you feel like you were nothing even though you're the one that was carrying the place and doing everything. P11 (Female, Senior support worker)

### 3.4 Summary of an explanatory model

**Figure 2:**
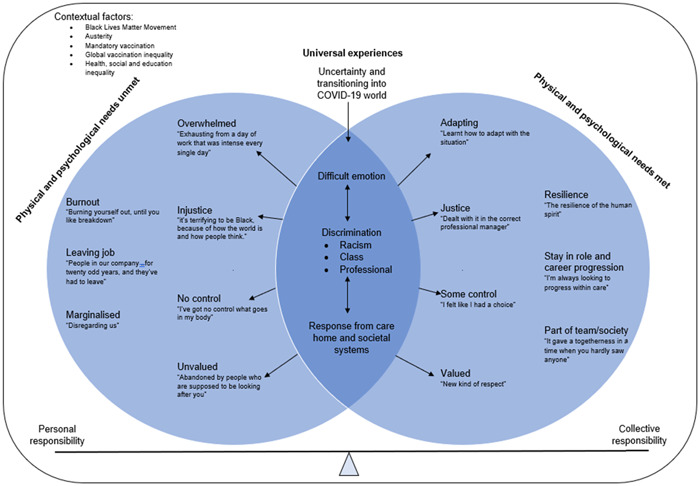
Grounded theory explanatory model of SoGM experiences of working during the pandemic and how this has influenced their identity. SoGM, Staff of the Global Majority.

Figure 2 depicts an explanatory model of the research findings, describing the dynamic and interconnected process participants experienced. The model conceptualises the emotional, social, and professional impacts working during the pandemic has had and how this has influenced their identity. The model is established within contextual factors described by participants such as global vaccination inequalities and mandatory vaccination.

The main elements of the model represented whether participants' physical and psychological needs were met or not. The overlap between needs being met or unmet highlights the universal experience shared by all participants and the influencing process involved in shaping participants' identities.

When physical and psychological needs were met, participants experienced a sense of being adaptable, having control, and being valued by others. Participants internalised this as part of their identity and expressed it as inner strength, and a sense of pride and professional skill. Similarly, a sense of justice, safety and a shared group identity developed when appropriate consequences and action were taken care home managers and the government. In comparison, when physical and psychological needs were not met, participants experienced a sense of being overwhelmed, unvalued and controlled by others. This led to feelings of weakness, burnout and feeling disconnected from others which led to increased job resignation.

Finally, a continuum of personal or collective responsibility relating to work practices and experiences during the pandemic demonstrates a balancing point. Acknowledge personal responsibilities but also recognise the wider system responsibility acted as a protective factor for identity.

## 4 DISCUSSION

This study explores the impact of the pandemic on the identity of SoGM working in care homes. The data revealed that the pandemic had negative effects on SoGM's emotional, psychological and physical well‐being. However, support from care homes and society mitigated these effects, indicating that meeting the physical and psychological needs of SoGM is crucial.

Overall, SoGM from care homes described a passion and desire to provide good quality care for residents however reported feeling frustrated, powerless and undervalued during the pandemic, leading to burnout and negatively impacting their identity. This aligns with similar findings on care home staff during the pandemic.^12^, ^27^ SoGM who experienced discrimination and felt a sense of inaction or injustice tended to describe feeling unvalued, accompanied by lower self‐esteem and job satisfaction. Schmitt et al.^28^ found that discrimination was associated with a decline in self‐esteem, life satisfaction, depression and anxiety. Racism and discrimination further compounded the challenges and fear experienced by staff, creating a sense of powerlessness and lower self‐esteem and job satisfaction.^29^ Discrimination was also found to be associated with a decline in self‐esteem, life satisfaction, depression and anxiety.^28^ Providing fair and supportive relationships in care homes and other social care services is crucial for allowing staff from ethnic minority backgrounds the opportunity to feel accepted, represented and valued. Incidents of discrimination, however, may have a less negative impact when experienced alongside a sense of shared identity or allyship, which is consistent with related research.^30^, ^31^ Similarly, SoGM also expressed an increased sense of collective identity as part of the ethnic minority group and profession due to their shared experiences of discrimination, becoming involved in collective action and support groups.

In line with the theory of intersectionality, to negotiate multiple identities by building links with various social networks, such as care home staff, family members and religious groups. Negotiating multiple identities appeared to form the basis for their new and strengthened identity, often noticing differences or similarities between these groups, for example, carers being seen as disposable and unvalued in society as well as individuals from minority ethnic backgrounds. Prioritising the NHS or ‘Us’ versus ‘them’ divisions resulted in SoGM feeling inferior, forgotten and reduced self‐worth. Strengthening professional and team identity connections is crucial for SoGM to access support and feel valued.

Furthermore, SoGM reported feeling stigmatised due to their ethnicity and job role, which is consistent with the research on the wider care home workforce.^12^ Ntontis et al.^32^ found that when individuals believe they are disproportionately impacted based on an existing social identity (e.g., being from a traveller background), this can impede identification with the new group due to the highlighted disparity between groups. For SoGM this stigma appears to be legitimised by the media and government which is consistent with similar research.^33^ Jetten et al.^34^ found that legitimised discrimination reduces collective identity and social support for stigmatised groups. Some SoGM expressed mistrust and dissatisfaction with the government's handling of the pandemic due to past experiences, misinformation and fear of repercussions. Legerski et al.^35^ found mistrust between affected communities and those in power can result in their needs not being met. Kadri et al.^36^ highlighted care home staff are seen as an instrument of care rather than independent persons with experiences, triggering a sense of being overlooked, consistent with SoGM's experiences. SoGM's negative experiences and the impact of public perception on their identity align with research showing care homes as undervalued.^37^, ^38^ To transform the public image of the care home sector and staff, government/local authorities must provide sufficient resources and funding.^27^

Consistent with social identity theory,^14^ SoGM's sense of belonging was influenced by their acceptance or rejection within groups. SoGM who had their needs met identified with a strong sense of resilience, practised self‐care more readily and maintained a positive sense of self by following their values and belief systems, demonstrating moral resilience. Research has found self‐efficacy and self‐control facilitates healthcare staff's ability to cope and respond effectively to distress.^39^ Moral resilience involves the staff's ability to maintain the courage and self‐confidence to face distressing and uncertain situations by following and trusting their values and belief systems. For SoGM this allowed them to maintain the balance between personal and collective responsibility and protect their positive sense of self. In line with research findings that indicated moral resilience prevents staff from viewing themselves as inadequate, futile, weak or neglecting others.^40^ Likewise, SoGM that experienced a sense of belonging, acceptance, being protected and being valued appeared to be more able to tend to and utilise self‐compassion, nurturing their well‐being. Effective self‐care strategies have been correlated with reduced levels of stress and caregiver burden.^41^ This self‐compassion helped SoGM to navigate ethical dilemmas and other workplace challenges by contextualising individual and systemic responsibility for resident care.

## 5 STRENGTHS AND LIMITATIONS

In line with grounded theory principles the researcher's position and background as a SoGM may have influenced the research design and participants' willingness to share their experiences. To reduce possible bias, the researcher used a reflective diary and discussions with the research team.

The study has limitations as the online recruitment strategy may have excluded some SoGM who had limited access to technology, and the majority of participants reported few COVID‐19‐related deaths. Attempts were made to recruit a wider variety of participants, but this was not feasible. As the participants had limited experience with COVID‐19 deaths, caution should be taken when generalising the findings to the wider SoGM population working in care homes. Future research should use randomly selected large samples to gain a clearer understanding of SoGM experiences and the mechanisms that impact their identity.

## 6 PRACTICE AND POLICY IMPLICATIONS

The study emphasised the negative impact of the pandemic and discrimination on staff's well‐being and identity. Care homes and adult social care organisations can improve their workplace cultures and team cohesion through support groups and psychological interventions that provide a space for staff to explore personal and ethical challenges. Peer support could incorporate a reflective space to share experiences and frustrations, like through Schwartz Rounds.^42^ Building a safer, more comfortable and constructive environment can promote staff retention and cohesion.^43^ The findings suggest that a collective identity is beneficial for well‐being and services should promote and maintain this where possible.

The study emphasised the importance of collective support when someone experiences discrimination. Organisations should take incidents seriously and resolve them while providing a collective support space. A culturally competent and informed workforce is necessary and can be achieved through staff training that enhances their cultural awareness, attitude toward cultural differences and cross‐cultural skills.

## 7 CONCLUSION

The pandemic has worsened the already challenging working environments in care homes, exposing structural deficiencies. All staff faced increased workloads and difficult emotions, but SoGM faced additional challenges like racism and discrimination. The lack of support and recognition for SoGM had a detrimental effect on their mental health, well‐being and identity, and the impact was influenced by individual and team responses. Additional support, training, access to support groups and strategies to enhance a collective identity in care homes and the adult social care sector are needed. Increasing shared and collective identities for all adult social care staff and the NHS could enhance staff personal identity, collaboration, staff retention and care quality.

## CONFLICT OF INTEREST STATEMENT

The authors declare no conflict of interest.

## Data Availability

The data that support the findings of this study are available from the corresponding author upon reasonable request.
